# Evaluation of Two Chemoenzymatic Glycan Remodeling Approaches to Generate Site-Specific Antibody–Drug Conjugates

**DOI:** 10.3390/antib12040071

**Published:** 2023-11-03

**Authors:** Qiang Yang, He Chen, Chong Ou, Zhihao Zheng, Xiao Zhang, Yunpeng Liu, Guanghui Zong, Lai-Xi Wang

**Affiliations:** 1 GlycoT Therapeutics, College Park, MD 20742, USA; 2 Department of Chemistry and Biochemistry, University of Maryland, College Park, MD 20742, USA; 3 ChemBind LLC, Atlanta, GA 30303, USA

**Keywords:** endoglycosidase, glycan oxazoline, chemoenzymatic glycan-remodeling, antibody-drug conjugates

## Abstract

Fc-glycosite-specific antibody–drug conjugation represents a promising direction for the preparation of site-specific antibody–drug conjugates (ADCs). In the present research, we conducted a systemic evaluation of two endoglycosidase-catalyzed chemoenzymatic glycoengineering technologies to prepare glycosite-specific ADCs. In the first two-step approach, the antibody was deglycosylated and then reglycosylated with a modified intact N-glycan oxazoline. In the second one-pot approach, antibodies were deglycosylated and simultaneously glycosylated with a functionalized disaccharide oxazoline. For the comprehensive evaluation, we first optimized and scaled-up the preparation of azido glycan oxazolines. Afterwards, we proved that the one-pot glycan-remodeling approach was efficient for all IgG subclasses. Subsequently, we assembled respective ADCS using two technology routes, with two different linker-payloads combinations, and performed systemic in vitro and in vivo evaluations. All the prepared ADCs achieved high homogeneity and illustrated excellent stability in buffers with minimum aggregates, and exceptional stability in rat serum. All ADCs displayed a potent killing of BT-474 breast cancer cells. Moving to the mouse study, the ADCs prepared from two technology routes displayed potent and similar efficacy in a BT-474 xenograft model, which was comparable to an FDA-approved ADC generated from random conjugation. These ADCs also demonstrated excellent safety and did not cause body weight loss at the tested dosages.

## 1. Introduction

The conjugation of various functional molecules to antibodies are frequently used and actively explored for a wide variety of applications within the life science sectors, such as fluorescent labeled antibodies for the detection and imaging [1], antibody–drug conjugates (ADCs) for cancer therapy [2,3,4,5,6,7], antibody–antibiotic conjugates for infectious disease treatment [8,9], antibody–immunostimulant conjugates for the treatment of cancer and other diseases [10], and lysosomal-targeting chimera (LYTAC) for the targeted degradation of proteins [11]. The need for stable, robust and controlled conjugation technologies are continuously growing within basic research, diagnostics, and therapeutic development [7,12,13].

The most notable and exemplified application of antibody conjugation is the development of ADC therapeutics. Over the past 10 years, ADCs have emerged as one of the most powerful and successful avenues for the treatment of cancer. By conjugating to targeted antibodies, highly toxic drugs are specifically delivered to tumor tissues to kill the cancer cells while largely sparing normal tissue [2,3,4,5,6,7]. Currently, 15 ADCs have been approved by the FDA and the regulatory agencies of other countries (Table 1), while about 200 other ADCs are being tested in various stages of human clinical trial towards different types of cancers (https://worldadc-europe.com/ (accessed on 1 March 2023)). For most of the ADCs that are on the market or currently in clinical trials, the payloads have been conjugated to antibody by non-specific random linkage to either cysteine or lysine, resulting in heterogenous ADC regioisomers, with varied antigen affinity, aggregation potential, serum half-life, and other limitations [7,14,15,16]. As a result, site-specific ADCs with more consistent quality attributes, improved pharmacokinetics, and an enhanced therapeutic index have been developed and evaluated in different stages [7,13]. Currently, a majority of the explorations and developments to achieve site-specific ADCs were based on protein engineering, such as engineered cysteines (ThioMab^TM^), enzyme-directed modification, and unnatural amino acid incorporation [7,13]. Another approach is conjugation through the Fc N-glycan at the conserved Asn-297 position to generate Fc-glycan-specific ADCs. The N297 position appears to be an optimal position for the site-specific drug conjugation of antibodies, as demonstrated in multiple preclinical reports. A Pfizer study indicated that among the various antibody locations (N-terminal of light chain, C-terminal of heavy chain, etc.), transglutaminase-catalyzed conjugation on the Q295 position, in close proximity to the glycosylation site, produced the ADC with the highest stability in incubation in mouse plasma, the best pharmacokinetic profile in mouse, and the most potent anti-tumor efficacy in a xenograft mouse model of pancreatic cancer [17]. In another study with GlycoConnect^TM^ technology [18], in vivo site-scanning of a glycan-specific conjugation demonstrated that the native N297 glycosylation site is the optimal conjugate position to achieve the best anti-tumor effect [18]. Finally, reports from a Merck research group and researchers from Binghamton University also corroborated that conjugation with Q295 demonstrated superior stability and pharmacokinetics to a wide range of other locations [19,20]. Multiple aspects of the advantages of site-specific Fc-glycan conjugation strategy can be explained: (1) Conjugation with Fc N-glycan, which is distant to the Fab region, usually would not interfere with the binding to the antigen; (2) The natural glycan linkage is usually stable in the blood circulation. In contrast, the popular cysteine-maleimide conjugation may incur retro-Michael additions, which would cause the detachment of linker-payload in the circulation process [21,22,23]; (3) The N-297 site, as a spatially hidden position, partially or fully shields the linker payloads to serum protease, depending on the length or chemical nature of the linker.

**Figure 1:**
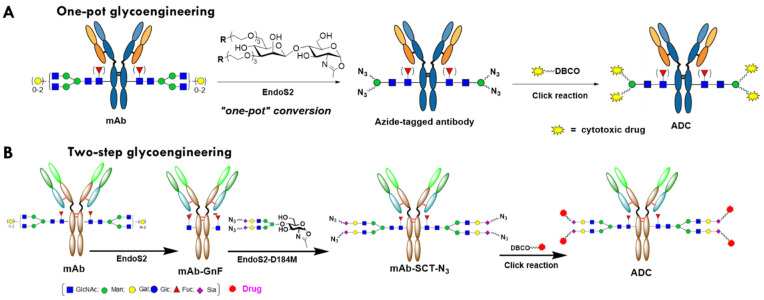
Schematic presentation of chemoenzymatic glycan remodeling for glycosite-specific antibody conjugation. SCT, sialylated complex-type glycan: N_3_, azide.

In recent years, different methods have been developed for Fc-glycan remodeling-based site-specific conjugation. Early attempts included the functionalization the Fc glycans through the oxidation of adjacent diols of terminal monosaccharides [24,25]. The use of the galactosyltransferase (GalT) mutants capable of accommodating modified UDP-galactose derivatives as the donor substrates enabled the incorporation of a selected tag at the terminal GlcNAc moieties on the Fc glycans for subsequent site-specific conjugation with modified cytotoxic agents [7,13]. The GlycoConnect technology [18,26], which comprises trimming Fc-glycan with endoglycosidase (Figure 1B) and the subsequent transfer of a GalNAz or an azido-Gal moiety and subsequent click of a payload [18,26], has been adopted by multiple clinical stage companies in the development of ADCs. However, there are limitations to this technology route. First, the Gal- or GalNAc-transferase (GalT or GalNAcT) is not a very efficient enzyme, requiring large quantities of enzymes (enzyme to substrate (E:S) weight ratio up to 1:20) and taking a prolonged time (overnight) to complete the full transfer [18,26]. Second, as the GalT or GalNAcT in this platform can only transfer azide- or keto-based small Gal-GDP derivatives, the conjugation loading points provided by this method is only 2 [18]. Third, the use of multiple enzymes may entail multiple purification steps in the process, which significantly increases the cost of the industrial manufactural process. In contrast, an endo-*N*-acetyl-β-D-glucosaminidases (ENGases)-based transglycosylation method recently developed by Wang and co-workers can overcome most of those limitations. As shown in Figure 1A, this approach utilizes the transglycosylation activity of an ENGase, EndoS2 from *Streptococcus pyogenes* of serotype M49, to transfer activated synthetic disaccharides with a conjugation tag (azide, etc.) to IgG in a single-enzyme, one-pot manner [27]. In this remarkable approach, the deglycosylation step (to trim out the heterogenous native glycan between two GlcNAcs within the chitobiose core) and the transfer of the azido disaccharide (in the form of activated glycan oxazoline) to the deglycosylated IgG occur simultaneously in the same reaction system, with just one enzyme. As the disaccharide is an unnatural substrate for EndoS2, the transglycosylation product of IgG-disaccharide-azide is highly resistant to hydrolysis by the enzyme due to the truncated modification. The azide-functionalized antibody can be subsequently conjugated with a functionalized payload through click reactions to produce a site-specific ADC [27]. Furthermore, it has been demonstrated that EndoS2 can also accommodate drug-preloaded disaccharide oxazoline derivatives as a substrate for transfer, enabling single-enzyme, one-pot ADC production [28,29].

On the other hand, another relevant two-enzyme glycan-remodeling approach has also been developed for the construction of ADCs. This approach consists of two enzymatic steps: deglycosylation of the antibody by an ENGase like EndoS2 to provide the precursor with the first N-acetylglucosamine (GlcNAc), with the core fucose still attached (antibody-GnF), and the subsequent attachment of a tagged (such as azide) biantennary sialylated complex-type (SCT) N-glycan to the deglycosylated antibody by a glycosynthase (ENGase mutant) such as EndoS-D233Q or EndoS2-D184M, which serves as the loading points for the functional molecules [30,31,32,33] (Figure 1B). The core enzyme of this platform, ENGase or glycosynthase, can transfer either disaccharide or a large glycan substrate, such as full-length N-glycan with terminal sialic acid, conjugated with the extended spacer-tag group [34,35]. In addition, either ENGase or glycosynthase can transfer the reaction with very high efficiency, and an E:S of lower than 1:300, and complete the reaction within two hours. This flexibility and efficiency are superior to other enzyme-based platforms to generate site-specific ADCs, such as GalT/GalNAcT (GlyConnect^TM^), sortase (SMAC^TM^) [36], transglutaminase [37,38], and farnesyltransferase (ConjuAll^TM^) [39]. The two-step glycan-remodeling technology (Figure 1B) was adopted by Daiichi Sankyo to develop its next-generation ADC. An anti-CLDN6 ADC with a pyrrolodiazepine derivative payload (DS-9606a) developed with this technology entered human phase 1 clinical trials in 2022 for the treatment of various solid tumors [40,41].

In early publications by Wang and co-workers [27,28], research works were focused on the glycan-remodeling, with limited in vitro characterization of generated ADCs. The ADC prepared was limited to trastuzumab, and the amount of produced and evaluated ADC was just a few milligrams. In current study, we further optimized, evaluated, and compared two glycosite-specific chemoenzymatic technology routes. We optimized and scaled-up the azido glycan oxazoline synthesis, prepared ADC with two different linker payload combinations, and performed systemic in vitro and in vivo evaluations of the azide-functionalized antibodies and the resulting site-specific ADCs. Our data indicate that the site-specific ADCs synthesized by the two Fc glycan remodeling methods have potent efficacy in cell-based assays and animal models, and they also have a high serum stability with less than 2% aggregation.

## 2. Results

### 2.1. Evaluation of the One-Pot Glycan-Remodeling Strategy for Different IgGs

**Figure 2:**
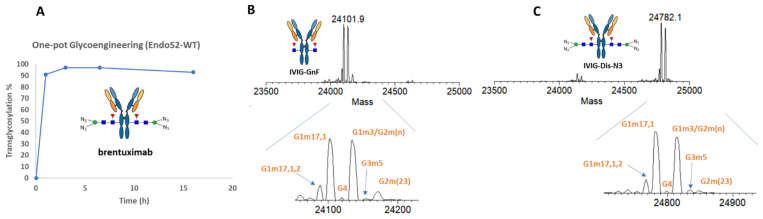
One-pot transglycosylation of brentuximab and IVIG. (**A**) Timecourse of transglycosylation of brentuximab. (**B**) LC-MS analysis of Fc/2 of deglycosylated IVIG generated by IDeS treatment [42]. (**C**) LC-MS analysis of Fc/2 of glycan-remodeled IVIG. The allotype peaks were assigned based on ref. [42].

For the one-pot transglycosylation, all research works to date only used trastuzumab as the model antibody [27,28,29]. To validate the applicability of the one-pot technology to other IgG1 mAb and other IgG subclasses, we tested the transglycosylation reaction with anti-CD30 brentuximab and intravenous immunoglobulins (IVIG), and pooled human antibodies containing IgG subclasses 1-4. As shown in Figure 2A, using the same condition established in ref. [27], also described in the experiment section, we achieved a 97% transfer of azido-disaccharide to brentuximab within two hours. The transglycosylation product did not show any hydrolysis by EndoS2 within 6 h. With overnight incubation at room temperature, only 3% of the product was deglycosylated by EndoS2, confirming the amazing deterrence of the product to EndoS2 hydrolysis (Figure 2A). Next, we tested if this EndoS2-catalyzed one-pot reaction was equally applicable to other subclasses of IgG by performing a transglycosylation study on IVIG, and a mixture of human IgG 1-4. As shown in Figure 2C, using the same conditions for monoclonal IgG1, the overall transfer rate achieved for IVIG was 92%. A careful examination of the ratio of different allotypes of IVIG between the deglycosylated IVIG (IVIG-GnF, Figure 2B) and the transglycosylation product (Figure 2C) did not show an apparent shift in the ratios among different allotypes of IgG, indicating that EndoS2 is capable of glycan-remodeling IgG1-4 subclasses with azide-disaccharide oxazoline of similar efficiencies.

### 2.2. Optimization of the Synthetic Routes for Azido SCT Oxazoline

**Figure 3:**
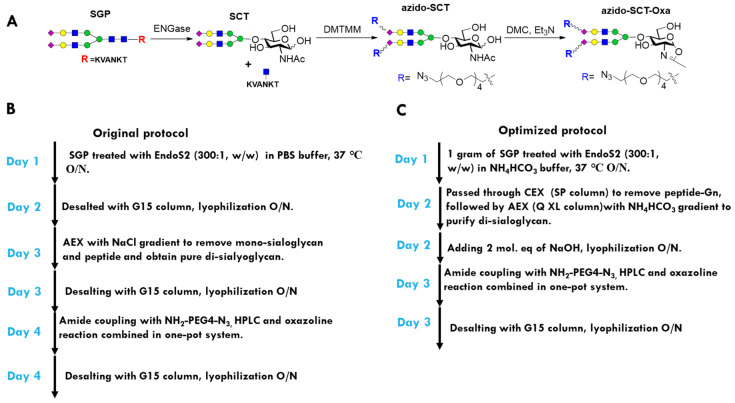
Optimization of the azido SCT oxazoline preparation protocol. (**A**) Scheme for preparation of the oxazoline from SGP. (**B**) Workflow of the original protocol. (**C**) Workflow of the improved protocol.

For the two-step Fc glycan remodeling route, the azide-functionalized disialyl complex-type glycan oxazoline is the key intermediate, and was synthesized using the sialoglycopeptide (SGP) as the starting material (Figure 3A) [43]. The synthetic steps from SCT to azido SCT oxazoline was optimized and reported by Wang and co-workers [35]. Nevertheless, the preparation of disalyl SCT from SGP is not optimal [35]. In the original protocol, to manufacture azido SCT-Oxa from SGP (Figure 3B), three desalting steps were used to remove the salts in EndoS2 digestion, NaCl from anion exchange (AEX) steps, and organic salt in the final oxazoline reaction. These desalting procedures, which require long and thin exclusion chromatography (SEC) column, posed a great challenge for scale-up and dramatically reduced the overall yield. To establish a new and scalable protocol, we used the ammonium bicarbonate (NH_4_HCO_3_) buffer in both enzyme digestions and AEX process, which eliminates the relevant desalting steps, as ammonium bicarbonate could be removed by lyophilization. We found that AEX on the HiTrap Q XL column with a 0–300 mM NH_4_HCO_3_ gradient could effectively remove monosialoglycan and undigested SGP (Figure S1). We also discovered a cation exchange (CAE) step with a HiTrap SP column that removes the positive peptide could improve the separation of mono- and disialylated glycan in the AEX procedure. With this improved protocol, we could produce 0.5 g of disial SCT from 1 g of SGP in just 3 days (Figure 3C). During the process, we also observed that residual ammonium (NH4^+^), after lyophilization, would react with the carboxyl group and resulted in 10–30% mono azido-SCT (Figure S2). This problem was readily addressed by the addition of 2 mol. eq. of sodium hydroxide before lyophilization. The sodium hydroxide neutralized the ammonium and the resulting sodium bicarbonate did not negatively impact the following azide-tagged-amine coupling and oxazoline reactions. As a result, a new protocol for preparing azido SCT-Oxa from SGP was established (Figure 3C), which reduced the SEC to only one time and could be proportionally scaled up to 100 grams, or even kilograms.

### 2.3. Preparation of Trastuzumab ADCs with Different Linker-Payload

**Figure 4:**
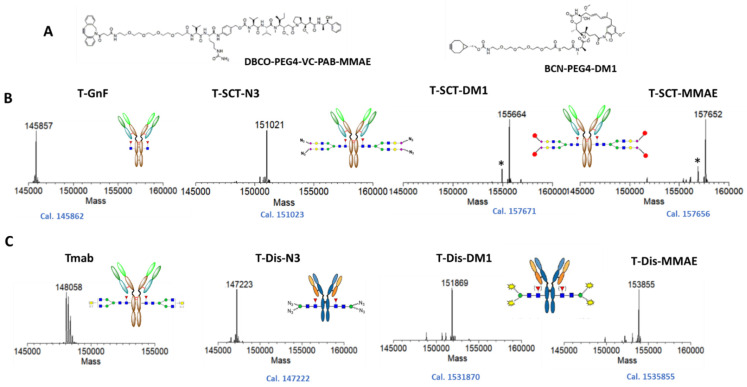
Preparation of glycosite-specific ADCs. (**A**) Chemical structures of linker-payloads with clickable groups. (**B**) Intact LC-MS analysis of preparation of ADC with two-step glycan remodeling. (**C**) Intact LC-MS analysis of ADC preparation with one-pot glycan remodeling. The measured M.W. was displayed on top of mass spectrum, while the calculation is shown below. * indicated ghost peaks generated by fragmentation in the LC-MS analysis.

After the optimization of the synthesis of azido glycan oxazolines, we employed two bioconjugation approaches and strain-promoted alkyne−azide cycloaddition (SPAAC) [44] to prepare glycosite-specific ADCs (Figure 1) with two different linker-payloads, an MMAE payload with a cleavable valine–citrulline linker, and maytansinoid derivate DM1 (also an inhibitor of tubulin polymerization) with a non-cleavable linker (Figure 4A). As the intermediates and final ADCs made using this chemoenzymatic glycoengineering approach are highly homogeneous, the whole process of glycan remodeling and SPAAC to assemble the ADCs could be directly and conveniently monitored with intact antibody LC-MS (Figure 4B,C). To prepare the ADCs with two-step remodeling, the trastuzumab (Tmab, further abbreviated as T) was deglycosylated with immobilized EndoS2, resulting in T-GnF, which was transglycosylated with EndoS2-D184M glycosythase to afford T-SCT-N_3_. Finally, T-SCT-N_3_ was conjugated with the commercially available linker-payload that was preassembled with clickable functional groups to generate the final ADCs (T-SCT-MMAE and T-SCT-DM1) (Figure 4B).

For synthesis of the ADCs using the azide–disaccharide substrate, we followed the procedures reported by Wang and co-workers [27]. In brief, first, we scale up the synthesis of azido-ManGlcNAc oxazoline to the 100 mg level. Subsequently, antibody Tmab was one-pot tranglycosylated with azido disaccharide (Dis) oxazoline to afford T-Dis-N_3_; then, it was conjugated with linker payloads to produce the final corresponding ADCs, T-Dis-DM1 and T-Dis-MMAE (Figure 4C). As shown in Figure 4B,C, the final prepared ADCs were highly homogenous, with a main peak of DAR 4.

### 2.4. In Vitro Characterization of the ADCs with Different Payloads

**Figure 5:**
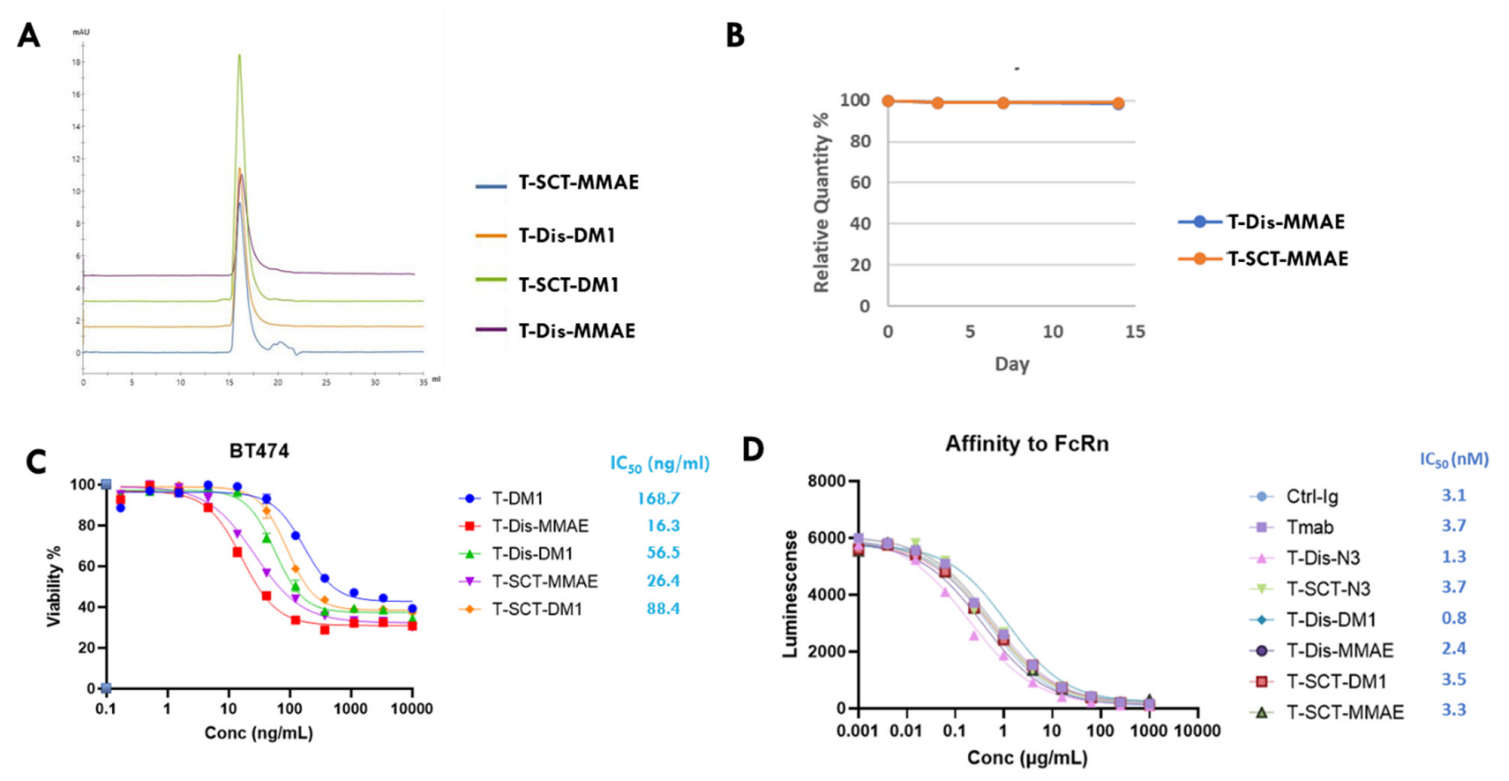
In vitro evaluation of glycosite-specific ADCs prepared with two glycan-remodeling approaches. (**A**) Aggregates analysis with size exclusion chromatography (SEC). (**B**) Rat serum stability. ADCs with cleavable linkers were incubated in rat serum at 37 °C for two weeks. The intergrity of ADCs at different timepoints was monitored with LC-MS. (**C**) Cytotoxicity of ADCs towards BT-474 breast cancer cells with high HER2 expression. Data points were presented as means ± SEM of triplicate measurements. (**D**) Affinities of ADCs, azido-functionalized Tmabs to FcRn at pH 6, measured with a Lumit^TM^ FcRn Binding Immunoassay kit. Data are means of duplicate measurements. SCT, sialylated full-length complex type glycan; Dis, disaccharide.

With four site-specific ADCs in hand, we performed a series of in vitro characterizations. First, we tested the stability and aggregation level of ADCs with size exclusion chromatography (SEC) analysis. All four ADCs contained less than 2% of aggregates in the PBS buffer, as analyzed with agarose-dextran particle-based SEC (Figure 5A). Similar results were obtained with another high-porosity silica-particle-based SEC analysis. ADCs were stable with 37 °C incubation for 7 days, without an increase in aggregation, as analyzed by SEC. We then tested the serum stability of the ADCs with cleavable linkers. As shown in Figure 5B, ADC with either disaccharides or full-length glycan was exceptionally stable in the rat serum, remaining intact for two weeks in 37 °C incubation. In contrast, ADCs with popular cysteine–maleimide conjugation generally lose half of their payloads in rat serum incubation assays due to the retro-Michael additional release of the maleimide group [21,22,23]. Subsequently, we performed a cytotoxicity study of ADCs on BT-474, an HER2-overexpressing breast cancer cell line. As shown in Figure 5C, all four glycosite-specific ADCs (gADCs) demonstrated a potent and similar killing to the BT-474 cells, with IC_50_ below 100 ng/mL. The gADCs with DM1 payloads demonstrated a slightly stronger cancer cell-killing ability compared to T-DM1 (Kadcyla^®^), an FDA-approved ADC with the same payloads and a similar DAR. Our final in vitro characterization of the gADCs was their affinity with the FcRn, the serum half-life determinant of native or conjugated antibodies. Using a Lumit^TM^ FcRn Binding Immunoassay kit, we determined that the affinities of four gADCs and two azido-functionalized mAbs are very similar to those of trastuzumab (Figure 5D). This is expected, as the interaction area between the antibody and FcRn is found on the interface between CH2 and CH3 domains of Fc, which is somewhat distant from the N297 glycosylation site.

### 2.5. In Vivo Characterization of Glycosite-Specific ADCs with Different Payloads

**Figure 6:**
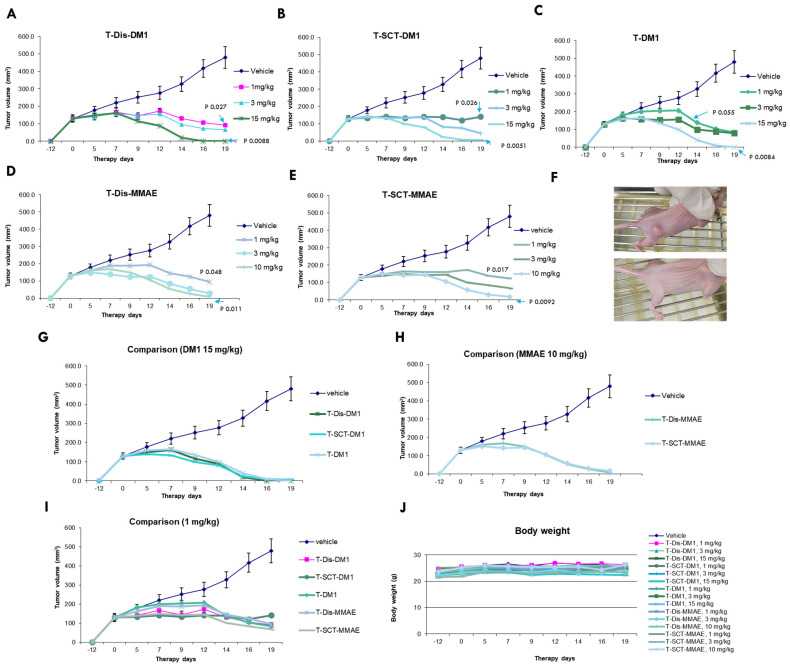
In vivo evaluation of glycosite-specific ADCs. (**A**–**E**) Dose responses of ADCs (*n* = 6). DM1 ADCs were administrated once at day 0 with 1, 3, and 15 mg/kg, while MMAE ADCs were administrated once at day 0 with 1, 3, and 10 mg/kg. Statistical analysis was performed with a two-tailed Welch’s t-test. Data points are presented as means ± SEM. (**F**) Representative tumor images of untreated and eradicated. (**G**–**I**) Comparison of different ADCs in the same dosage (*n* = 6). Data points are presented as means ± SEM. (**J**) Body weight curves of control and treatment groups. No significant bodyweight loss was observed for any treatment group.

After the systemic in vitro characterizations, we tested the gADCs with two different linker-payloads in a BT-474 xenograft mouse model, with side-by-side comparison to FDA-approved T-DM1. All ADCs were intravenously administered using a single injection with various dosages. The tumor growth and mice body weight were continuously monitored for three weeks. As shown in Figure 6, all ADCs showed a potent dose-dependent inhibition of tumor growth (Figure 6A–E). At a dosage of 15 mg/kg for DM-1 ADC and 10 mg/kg for MMAE ADC, the one-time IV injection of the ADC induced tumor eradication (Figure 6A–F). A lower dose, even at 1 mg/kg, still caused significant tumor shrinkage or growth inhibition (Figure 6A–E). The ADCs prepared using two different routes demonstrated almost identical anti-tumor efficacies, with different linker-payloads. Compared to non-specific conjugated T-DM1, glycosite-specific ADCs demonstrated comparable efficacies at the highest dosage (Figure 6G,H). At a low dosage of 1 mg/kg, glycosite-specific ADCs illustrated slightly better activity compared to T-DM1 (by day 12), although the differences are not statistically significant (Figure 6J). At the tested doses, no ADCs caused apparent weight loss in mice (Figure 6K).

## 3. Discussion

In the present study, we performed a systemic evaluation of two glycosite-specific chemoenzymatic antibody–conjugation technology routes for the preparation of site-specific ADCs. We optimized and scaled-up the preparation of azido glycan oxazolines. The optimization and scale-up of azido-SCT oxazoline was very successful to a 500 mg level, and can be further scaled-up proportionally. The synthesis of azido-ManGlcNAc oxazoline was also scaled-up to 100 mg in the current study.

After optimization of the synthesis of azido glycan oxazoline, we assembled ADCs with two technology routes, with two different linker-payloads combinations (DAR of 4), and performed systemic in vitro and in vivo evaluations. All ADCs illustrated excellent stabilities in buffers with minimum aggregates, exceptional stability in rat serum at 37 °C for two weeks, and potent killing of BT-474 breast cancer cell lines. The potency of DM1 glycosite-specific ADCs is comparable with randomly lysine-conjugated and T-DM1 When comparing the payloads, MMAE ADCs are more powerful than those of DM1. In the final in vitro test, the ADCs showed a similar affinity with the FcRn receptors as that of trastuzumab, which indicated that the gADC may maintain a long serum half-life in vivo, at a similar level to its antibody precursors. Moving to the animal study, the ADCs produced using the two technology routes displayed a very potent and similar efficacy in a BT-474 mouse xenograft model. The ADCs did not cause body weight loss at 15 mg/kg, the highest test level. The MMAE gADCs with a cleavable linker showed similar levels to DM1 gADCs and randomly conjugated T-DM1, which is slightly distinct from the in vitro results. The BT-474 model we used in the current study is a strong, positive HER2 model, in which the bystander effect of the MMAE payload with a cleavable linker may not contribute to the whole anti-tumor effect. When comparing glycosite-specific versus randomly conjugated ADC, DM1 gADCs demonstrated similar anti-tumor activity compared to T-DM1, which carries the non-cleavable linker.

During the course of this study, Huang and co-workers published a comprehensive study of glycosite-specific ADCs with different linker configurations [45]. The study by Huang’s group is more focused on the one-pot preparation of glycosite-specific ADC with disaccharides oxazoline preassembled with a linker-payload. One interesting observation from the Huang group is that while disaccharide-derived ADCs displayed reduced interaction with Fcγ receptors, intact glycan-derived gADCs retained full affinity with Fcγ receptors. This suggested that the two-step glycan remodeling approach may be well-suited to the development of immunostimulatory antibody–drug conjugates (iADC) [46], in which the interaction between iADC and Fc Fcγ receptors on the immune cells has to be retained. It should be pointed out that while the one-pot synthesis of the ADCs with the direct use of drug-preloaded disaccharide oxazoline as the substrates appears to be more straightforward [28,29,45], the enzymatic conjugation efficiency using the drug-loaded disaccharide oxazolines is much slower than the corresponding azide–disaccharide enzymatic conjugation method [28], requiring much more enzyme and a much longer incubation time to complete the conjugation. As a result, scaling up the production of the ADCs could be challenging.

In summary, in this study, we evaluated and demonstrated the remarkable efficiencies and scalabilities of two chemoenzymatic glycan remodeling approaches for the development of site-specific ADCs. The excellent in vitro and in vivo properties of the prepared ADCs manifested in this research warrants further application of two approaches for the development of therapeutic ADCs.

## 4. Materials and Methods

### 4.1. Reagents and Materials

Trastuzumab (Herceptin), T-DM1 (Kadcyla), and IVIG were purchased from RefDrug (Hillsborough, NJ, USA). Brentuximab was produced by GeneMeta (German Town, MD, USA). Egg yolk powder was bought from BulkFoods (Toledo, OH, USA). DBCO-PEG4-VC-PAB-MMAE and BCN-PEG4-MCC-DM1 were acquired from BroadPharm (San Diego, CA, USA). Other chemicals, reagents, and solvents were purchased from Sigma-Aldrich and/or TCI, and used as-received unless otherwise specified.

### 4.2. Expression and Purification of Enzymes

EndoS2 and EndoS2-D184M was produced according to ref [47]. IgG-specific protease IDeS was prepared as described in ref. [48].

### 4.3. Liquid Chromatography Electrospray Mass Spectrometry (LC-ESI-MS) 

LC-MS analysis of glycans, glycopeptides and payload were performed on a HPLC-SQ2 system (Waters, Milford, MA, USA) with a C18 column (XBridge C18, 2.1 × 50 mm, 3.5 µm, Waters) using water containing 0.1% formic acid as phase A, acetonitrile containing 0.1% formic acid as phase B. LC-MS analysis of intact antibody was conducted with on a Q-Exactive OrbiTrap system (ThermoFisher, Waltham, MA, USA) with a C4 column (Advantage 300+ C4, 2.1 × 50 mm, 5 μm, Analytical Sales and Services) with 5–90% acetonitrile (containing 0.1% formic acid) linear gradient. The raw data were deconvoluted with MagTran (Amgen). The IDeS-treated IgG was analyzed with C8 column (Poroshell 300SB-C8, 1.0 × 75 mm, 5 μm, Agilent) with 25–35% acetonitrile (containing 0.1% formic acid) gradient. The raw data were deconvoluted with MagTran.

### 4.4. Synthesis of Azido SCT Oxazoline

Sialylated complex type (SCT) glycan was prepared from sialylglycopeptide (SGP) that was isolated from egg yolk powder [49]. In a typical protocol, 1 g of SGP was digested with EndoS2 in 1:200 ratio (*w*/*w*) in 5 mM ammonium bicarbonate (NH_4_HCO_3_) buffer, pH 7.8 at 37 °C overnight to dissociate the glycan and peptide portions. After confirmation of the cleavage with LC-MS, the digestion mixture was passed through a cation exchange column (HiTrap SP HP, 5 mL, Cytiva, Marlborough, MA, USA) that removes the positively charged peptide. Afterwards, the material was purified with anion exchange on two tandemly connected HiTrap Q XL column (5 mL, Cytiva) with a 0–300 mM NH_4_HCO_3_ gradient which can effectively remove monosialoglycan and undigested SGP. The purified SCT fraction was added to 2 mol. eq. of NaOH and then lyophilized. The dried SCT was functionalized with amino-PEG4-azide and converted to oxazoline following the reported protocol [35].

### 4.5. One-Pot Glycoengineering of Brentuximab and IVIG

A total of 1 mg of brentuximab or IVIG was mixed with 40 mol. eq. of azido Man-GlcNAc oxazoline, 1 µg EndoS2 in 50 µL of 50 mM NaPO4 buffer, pH 7.0, and incubated at room temperature. Aliquots of the reaction mixture were collected at indicated timepoints, treated with IDeS, and subjected to LC-MS analysis.

### 4.6. Preparation of Trastuzumab gADCs with Different Linker-Payloads

The one-pot transglycosylation of trastuzumab (Tmab) with azido Man-GlcNAc oxazoline was performed with linear enlargement to 50 mg, following the procedure described in the above paragraph, and then purified with protein A affinity chromatography. Subsequently, the azido Dis-Tmab was conjugated with either BDCO-PEG4-VC-PAB-MMAE or BCN-pEG4-MCC-DM1 though SPAAC [44], following published procedures [27]. The final ADC was purified with protein A affinity chromatography and buffer changed to PBS. The preparation of azido-SCT functionalized and conjugated ADC with same linker-payloads was conducted following the procedure described in ref. [35], with linear enlargement.

### 4.7. SEC Analysis

The aggregation levels of four ADCs were determined with size exclusion chromatography (SEC) on two different types of SEC column. For an agarose–dextran-based Superdex^®^ 200 Increase 10/300 GL column (Cytiva) column, 50 μg of each ADC was injected and analyzed on AKTA pure FPLC system with PBS as the mobile phase. In another SEC analysis, 5 μg of each ADC was loaded on to a high-porosity silica particle-based column (AdvanceBio SEC, 4.6 × 150 mm, 300 Å, 2.7 μm, Agilent (Santa Clara, CA, USA) and analyzed with analytical Vanquish HPLC system (Waters) with 150 mM NaPO4, pH 7.0 as the mobile phase.

### 4.8. Rat Serum Stability Test

The tests were conducted according to the procedures reported in ref. [23]. Aliquots of ADC serum mixtures were taken at indicated timepoints, purified with anti-human IgG-Fc agarose slurry (Sigma Aldrich), and analyzed with LC-MS.

### 4.9. Affinity to FcRn Receptors

The affinities glycan–remodel mAb intermediate and gADCs to FcRn were assayed with a Lumit^TM^ FcRn Binding Immunoassay kit from Promega, following the manufacturer’s manual. The assay was conducted in doublets and the fluorescence was measured with a multiplex mode microplate reader.

### 4.10. Cancer Cell Killing Assay

BT-474 (ATCC, Manassas, VA, USA) cells were cultured in Hybri-Care medium (ATCC), supplemented with 10% FBS, 50 U/mL penicillin, and 50 μg/mL streptomycin (ThermoFisher, St. Louis, MO, USA), seeded at 20,000 cells/well into a 96-well plate and grown overnight until 60% confluent. Cells were then washed with PBS and incubated with fresh media containing the ADCs, starting at a concentration of 1 μg/mL and serially diluted at a ratio of 1:2. Each compound was assessed in duplicate wells, and cells without a compound served as control. Plates were incubated for 72 h and cell viability was analyzed by CellTiter-Glo Luminescent Cell Viability Assay (Promega, Madison, WI, USA) as per manufacturer’s instructions. In brief, a volume kit reagent equal to the media was added to each well. Cells were then lysed and incubated for 10 min before luminescence was recorded with a microplate reader.

### 4.11. Xenograft Mouse Studies

The animal study was contracted to Washington Biotechnology Inc. (Baltimore, MD, USA). To establish the subcutaneous tumor xenograft, 2 × 10^7^ BT-474 cells containing Matrigel (50:50, *v*:*v*, BD Biosiences) were inoculated in the right flank subcutaneously into 5-week-old athymic nude mice. When the average tumor size reached approximately 150 mm^3^, the mice were injected intravenously with the indicated doses of ADCs. The tumor sizes and weight of mice were continuously monitored for three weeks after the initial injection of ADC.

## Figures and Tables

**Table 1:** ADCs approved by global regulatory agencies.

Company/Partner (Trade Mark)	Common Name	INN	Indications	Approval	Target	Antibody Conjugation	DAR	Linker	Payload
Pfizer (Mylotarg)	CDP-771	Gemtuzumab ozogamicin	acute myeloid leukaemia (AML)	2000/2017 (USA)	CD33	IgG4 (Lysine)	2~3	Cleavable hydrazone-disulfide	Calicheamicin
Seagen/Takeda (Adcetris)	SGN-35	Brentuximab vedotin	Hodgkin’s Lymphoma (HL) and anaplastic large-cell lymphoma (ALCL)	2011 (USA)	CD30	IgG1 (Cysteine)	~4	Cleavable Val-Cit	MMAE
Genetech (Kadcyla)	T-DM1	Trastuzumab emtansine	HER2 + metastatic breast cancer	2013 (USA)	HER2	IgG1 (Lysine)	3.5	Non-cleavable SMCC	DM-1
Pfizer (Besponsa)	CMC-544	Inotuzumab ozogamicin	acute lymphoblastic leukaemia (ALL)	2017 (USA)	CD22	IgG4 (Lysine)	~4	Cleavable hydrazone-disulfide	Calicheamicin
Astrazeneca (Lumoxiti)	CAT-8015	Moxetumomab pasudotox	hairy cell leukemia (HCL)	2018 (USA)	CD22	Fc/2 Ab	1	Fusion protein	PE38
Genetech (Polivy)	DCDS4501 /RG7596	Polatuzumab vedotin	diffuse large-B-cell lymphoma (DLBCL)	2019 (USA)	CD79b	IgG1 (Cysteine)	3.5	Cleavable Val-Cit	MMAE
Daiichi Sankyo (Enhertu)	DS-8201/T-DXD	Trastuzumab deruxtecan	HER2+ metastatic breast cancer	2019 (USA)	HER2	IgG1 (Cysteine)	7.6	Cleavable GGFG	DXD
Seagen/Astella (Padcev)	ASG-22ME	Enfortumab vedotin	Metastatic urothelial cancer	2019 (USA)	Nectin-4	IgG1 (Cysteine)	3.8	Cleavable Val-Cit	MMAE
Immunomedics/Gilead (Trodelvy)	IMMU-132	Sacituzumab govitecan	Triple negative breast cancer	2020 (USA)	TROP2	IgG (Cysteine)	7.6	Cleavable CL2A	SN38
GSK (Blenrep)	GSK2857916	belantamab mafodotin	Multiple myeloma	2020 (USA) *	BCMA	IgG1, (Cysteine)	~4	Non-cleavable mc	MMAF
Rakuten (Akalux)	RM-1929	Cetuximab sarotalocan	Head and neck squamous cell carcinomas (HNSCC)	2020 (Japan)	EGFR	IgG1 (lycsine)	~3	Non-cleavable	IR700
ADCT (Zynlonta)	ADCT-402	Loncastuximab- tesirine	Diffuse Large B-cell lymphoma	2021 (USA)	CD19	IgG1 (Cysteine)	2.3	Cleavable Val-Ala	PBD dimer
Seagen (Tivdak)	TF-011-MMAE	Tisotumab vedotin	Recurrent or metastatic cervical cancer	2021 (USA)	Tissue factor	IgG1 (Cysteine)	4	Cleavable Val-Cit	MMAE
RemeGen (Aidixi)	RC48	Disitamab vedotin	gastric cancer	2021 (China)	HER2	IgG1 (Cysteine)	4	Cleavable Val-Cit	MMAE
Immunogen (ELAHERE)	IMGN-853	Mirvetuximab soravtansine	Platinum-Resistant Ovarian Cancer	2022 (USA)	FRa	IgG1 (lysine)	3.4	Cleavable Sulfo-SPDB	DM4

Abbreviations: BCMA, B-cell maturation antigen; DM1, mertansine; DXD, deruxtecan; FRa, folate receptor a; INN, international nonproprietary name; mc, maleimidocaproyl; MMAE, monomethyl auristatin E; MMAF, monomethyl auristatin F; PBD, pyrrolobenzodiazepine; SMMC: *N*-succinimidyl-4-(*N*-maleimidomethyl) cyclohexane-1-carboxylate; SPDB, *N*-succinimidyl 4-(2-pyridyldithio) butyrateTROP2, tumour-associated calcium signal transducer 2. * Blenrep was withdrawn from market in November 2022.

## Data Availability

The dataset supporting the conclusions of this article is included within the article.

## Supplementary Materials

The following supporting information can be downloaded at: https://www.mdpi.com/article/10.3390/antib12040071/s1, Figure S1: (A) LC-MS analysis of reaction product of SGP digested by EndoS2. (B) Carbohydrate staining of AEX separation of di- and mono-sialyl glycans. (C) LC-MS analysis of purified disalylglycan; Figure S2: Molecular structure of mono azido-SCT.
