# Supplementary material for: Evaluation of Two Chemoenzymatic Glycan Remodeling Approaches to Generate Site-Specific Antibody–Drug Conjugates
Source: Antibodies (Basel). 2023 Nov 3;12(4):71. doi: 10.3390/antib12040071 (PMC10660516; doi:10.3390/antib12040071)
Supplement: Supplementary file 1 [file antibodies-12-00071-s001.zip › antibodies-2639737-supplementary.pdf]

Evaluation of two chemoenzymatic glycan remodeling approaches to generate site-specific antibody-drug conjugates

Qiang Yang[a*], He Chen[a], Chong Ou[b], Zhihao Zheng[a], Xiao Zhang[b], Yunpeng Liu[c], Guanghui Zong[b], Lai-Xi Wang[b*]

a GlycoT Therapeutics, College Park, MD 20742; b Department of Chemistry and Biochemistry, University of Maryland, College Park, MD 20742; c ChemBind LLC, Atlanta, GA

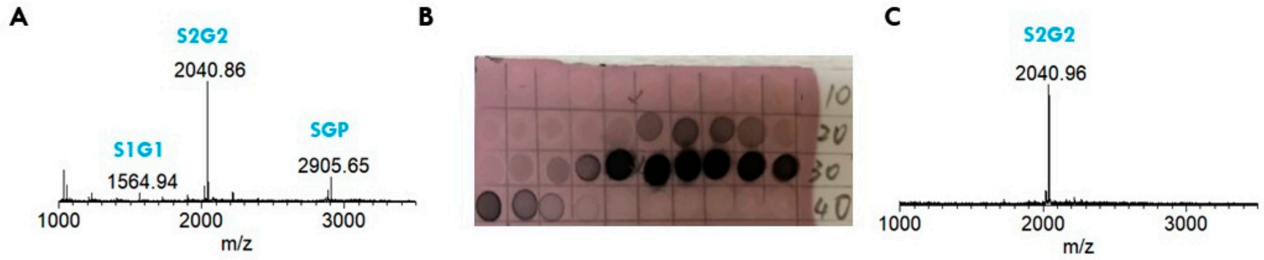

Supplementary Figure S1. A) LC-MS analysis of reaction product of SGP disgested by EndoS2. B) Carbohydrate staining of AEX seperation of di- and mono-sialyl glycans. C) LC-MS analysis of purified disalylglycan

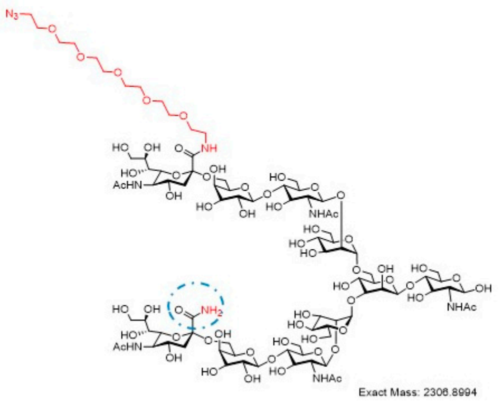

Supplementary Figure S2. Molecular strucutre of mono azido-SCT
